# Multiple sclerosis therapy consensus group (MSTCG): answers to the discussion questions

**DOI:** 10.1186/s42466-021-00140-1

**Published:** 2021-08-06

**Authors:** Heinz Wiendl, Ralf Gold, Frauke Zipp, for the Multiple Sclerosis Therapy Consensus Group, Thomas Berger, Florian Deisenhammer, Franziska Di Pauli, Christian Enzinger, Elisabeth Fertl, Michael Guger, Fritz Leutmezer, Orhan Aktas, Karl Baum, Martin Berghoff, Stefan Bittner, Achim Gass, Klaus Gehring, Norbert Goebels, Ralf Gold, Aiden Haghikia, Hans-Peter Hartung, Fedor Heidenreich, Olaf Hoffmann, Boris Kallmann, Christoph Kleinschnitz, Luisa Klotz, Verena Leussink, Volker Limmroth, Ralf Linker, Jan D. Lünemann, Mathias Mäurer, Sven G. Meuth, Uta Meyding-Lamadé, Michael Platten, Peter Rieckmann, Stephan Schmidt, Martin Stangel, Hayrettin Tumani, Martin S. Weber, Frank Weber, Heinz Wiendl, Uwe Zettl, Tjalf Ziemssen, Frauke Zipp, Andrew Chan, Adam Czaplinski, Tobias Derfuss, Renaud Du Pasquier, Claudio Gobbi, Andreas Lutterotti

**Affiliations:** 1 Klinik für Neurologie mit Institut für Translationale Neurologie, Westfälische Wilhelms-Universität Münster, Münster, Germany; 2 Neurologie, St. Josef-Hospital/Ruhr-University Bochum, Bochum, Germany; 3 Klinik und Poliklinik für Neurologie, Universitätsmedizin der Johannes Gutenberg-Universität Mainz, Mainz, Germany

## Question 1: when should disease-modifying immunotherapy be initiated? Before or after the onset of disability? Already with CIS (e.g., isolated optic neuritis)?

An explicit goal of multiple sclerosis (MS) therapy is the “best possible disease control”, including the “best possible quality of life” of the patient, with the option to use highly effective therapeutics early or as early as possible in response to disease activity. Specifically, the appropriate disease-modifying therapy (DMT) is selected **based on the individual patient**, and incorporates a situation and prognostic analysis that includes disease activity, disease severity, balancing therapy safety and risks, and considering the patient’s age, gender, and living situation. From the perspective of the MSTCG (Multiple Sclerosis Therapy Consensus Group) and supported by several large observational and registry studies, modern MS therapy can and should **prevent the accumulation of disability** and, thus, possible neurodegeneration.

**Fig. 1:**
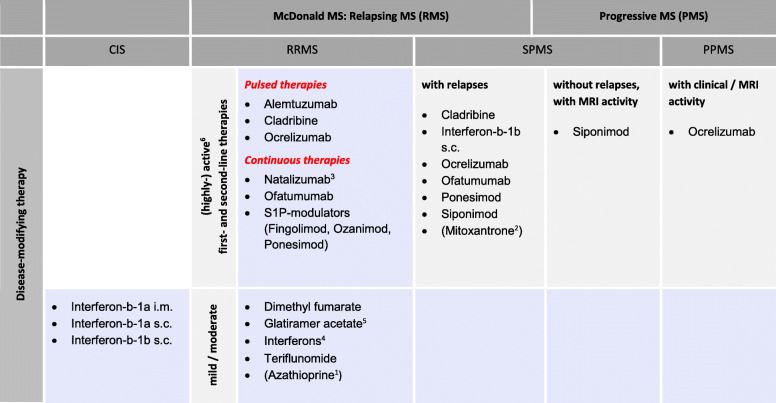
Disease-Modifying Therapy of MS. 1 - Azathioprine is formally approved but rarely applied (2nd choice); 2 - Mitoxantrone formally approved here as well as in highly active RRMS but rarely applied due to the unfavorable side effect profile and the cumulative maximum dose (2nd choice); 3 - Natalizumab: both i.v. and s.c.; especially in case of HPyV-2 (JCV) antibody positivity (HPyV-2 [JCV] Ab ≥0.9 HPyV-2 [JCV] Ab titer) risk stratification is essential due to PML risk! High risk for PML after i) prior immunosuppression, ii) ≥ 18 months of continuous therapy, and with iii) positive HPyV-2 (JCV) Ab status; 4 - Interferons: interferon-b-1a i.m., interferon-b-1a s.c., interferon-b-1b s.c., pegylated interferon-b-1a s.c./i.m.; 5 - Glatiramer acetate includes other glatiramoids. 6 - Decisions on type of therapy (as well as therapy concept) depend on the level of disease activity and severity; thus first- and second-line therapies are included here. Available drugs are listed alphabetically, not by strength or preference. Scheme from: MSTCG, DGNeurology Kommentar (2021) 10.1007/s42451-021-00353-3

The MSTCG recommends classifying MS as mild/moderate or active/highly active (see Fig. 1). The classification is based on i) relapse frequency, ii) MRI findings (lesion load, lesion localization), and iii) regression of relapse(s), disease activity, and disease severity (measured by clinical as well as radiological parameters); also, the patient’s age and comorbidities have to be considered. Activity is determined based on clinical relapses (severity of clinical symptoms/duration/tendency to regress) and/or MRI activity (contrast-enhancing lesions; new or enlarged T2 lesions). Progression is determined by an annual or more frequent examination, including a careful clinical assessment. In addition to the EDSS, standardized instruments for assessing clinical function in patients with MS include the Multiple Sclerosis Functional Composite (MSFC), the Brief International Cognitive Assessment for MS (BICAMS), the 6- and 2-min walk tests, or the timed 25-ft walk test. Notably, the MS classifications are not categorical and rigid: they require continuous review and close follow-up.

It is generally agreed that DMTs have a better effect early in the course of MS. Recent registry data indicate that later initiation of DMT leads to more extensive disability in the longer term [1–3]. In addition to preventing acute episodes of the disease, prophylactic therapy may reduce the risk of long-term neurologic deterioration or secondary progression [4]. The long-term therapeutic benefits strongly depend on how early DMT is started.

Due to the great heterogeneity of the clinical MS course, the further individual patient’s course is extremely difficult to predict. Although the term “benign MS” has been abandoned eventually, there may be courses that do not lead to any (significant) disability after 30 years – even without therapy. While in overall analyses up to 15–20% of patients may not accumulate measurable disability in the longer term, there are no reliable or accepted predictors for a course without substantial disability. Hence, DMT in MS must be started as early as possible after diagnosis to avoid further/future disability. In individual cases, a wait-and-see approach with regular neurological and imaging checks may also be considered in patients with very low lesion burden and complete remission of mild clinical symptoms.

In the context of a clinically isolated syndrome (CIS) suggestive of central nervous system demyelination, defined as a monofocal or multifocal clinical event, e.g., optic neuritis or a spinal cord, brain stem, or hemispheric syndrome, the MSTCG takes a proactive position on the management of these patients. Currently, three licensed medical treatments are available for CIS patients (Interferon-b-1a i.m., Interferon-b-1a s.c., Interferon-b-1b s.c.). (Interestingly, in the USA, siponimod is a recently approved additional option). Furthermore, it is of utmost importance that in this early phase of the disease, the affected patients become aware of and compliant to the need to monitor the situation to ensure the best possible treatment and management in the long term to prevent disease activity and neurodegeneration. This includes clinical and MRI follow-up and patient education in this specific phase of the disease. In persons with isolated optic neuritis without further evidence of CNS pathology on MRI, there is the possibility of no further progression to MS. Hence, here disease monitoring without treatment can be an option to consider in discussion with the patient [5]. In patients with further evidence of CNS lesions or oligoclonal bands that do not fulfill the MRI criteria for a full MS diagnosis, medical therapy needs to be clearly favored and advised over a pure monitoring approach. Any decision-making is a joint process requiring a responsible choice by the affected patient. In case of no immediate medical treatment, disease monitoring will be continued to uphold the opportunity for an effective early therapeutic intervention.

Generally, under exclusion of other differential diagnostic causes, CIS patients (regardless of whether the criteria for DIT and DIS are met) must be offered immunotherapy. The choice of immunotherapy should be based on predictive parameters; primarily i) MRI findings (number and localization of lesions) but also ii) extent of relapse regression, iii) multifocal presentation, and iv) CSF-specific OCB or chronic inflammatory CSF changes. For CIS patients with a high lesion burden and/or infratentorial lesions on diagnostic MRI, immunotherapy should be actively recommended given the presumed unfavorable prognosis. Here, depending on the individual circumstances, high-efficacy therapy can be considered already for initial treatment. Importantly, the treatment of CIS should not be unnecessarily delayed and should not follow an escalation approach in the individual (highly-active) case.

Last but not least, already CIS can be considered as MS in several colleagues’ opinions. The latest revision of the McDonald criteria allows the diagnosis of MS in many cases previously considered as CIS [6, 7].

## Question 2: which disease-modifying therapy should be selected when? Should a less potent and low-risk therapy initially be selected for all patients, or should some patients immediately receive a high-efficacy therapy with a higher risk profile and requiring more complex monitoring? To be considered in this context: escalation in three stages versus individually adjusted escalation

Presently, two treatment approaches dominate the selection of optimal therapy for (highly) active MS. Both strategies are based on evaluating the individual patient’s risk of further MS progression and considering the risk versus efficacy of the specific disease-modifying therapies. According to the escalation approach, lower-efficacy therapies with a known and relatively safe risk profile are selected for initial treatment. If – despite sufficiently long and regular treatment – disease activity persists/recurs, treatment is escalated to a more potent therapy option. In the alternative approach, treatment is initiated with a high-efficacy disease-modifying therapy already at the time of diagnosis; for example, with alemtuzumab, cladribine, natalizumab, ocrelizumab, ofatumumab, or S1P receptor modulators (fingolimod, ozanimod, ponesimod).

Limiting current treatment recommendations to the escalation approach is insufficient regarding the current data situation and diminishes the possibilities to start treatment with a higher-efficacy medication.

The DGN guideline attempts to grade disease-modifying therapies according to potency and thus divides medications into three groups. In the view of the MSTCG, this is scientifically unsound as any minimal differences in the percentage of relapse reduction cannot be formally compared between the drugs due to different study collectives. Problematically, this scientifically not well-founded approach ultimately results in a “three-part division” of the therapy algorithm – consisting of first a low-potent, then a medium-potent, and finally a high-potent therapy. This stepwise escalation, which is described as the preferential approach, does not correspond to established consensus recommendations, new study findings, or European/international therapy concepts – according to which not only the time of therapy (earliest possible) but also the potency of the therapy influences the long-term outcome. Thus the meanwhile more proactive therapy concepts and the freedom of therapy are ultimately restricted with a three-part escalation.

Choosing the first DMT in MS patients is challenging. The choice must occur on an individual patient level and take into account several factors: clinical symptoms, MRI activity, the efficacy of the therapeutic agent, side effects of the therapeutic agent, handling, route of administration, and the patient’s life circumstances and family situation [8, 9]. A general rule applies: the more potent the DMT, the higher the potential risk of severe side effects. The escalation regimen, in which therapy is always started with a less effective drug and switched to a high-efficacy DMT if disease activity persists, was initially advocated when only a few DMTs were available. With the availability of multiple high-efficacy DMTs, including depleting therapies, the hit-hard-and-early concept was postulated, recommending the use of high-efficacy DMT at disease onset, in analogy to, for example, rheumatology. Controlled trials that might demonstrate the superiority of one of these therapeutic approaches have now been initiated, but results will not be available for several years. Retrospective registry studies already suggest that in patients with disease activity, early use of high-efficacy DMT compared to lower-efficacy DMT may delay subsequent disability progression or transition to SPMS [2, 4]. The underlying reason may be in concordance with delaying therapy initiation early in the disease: persistent clinical or subclinical disease activity under less effective therapy may cause irreversible neurological deficits and allow the activation of signaling pathways associated with progressive disease that could have been otherwise prevented.

High-efficacy therapies are not suitable for every patient and require an individual risk-benefit assessment. Depleting or immune reconstitution therapies (IRTs), including autologous hematopoietic stem cell transplantation, have a special position in this regard. They cause profound changes in the immune system. Thus, on the one hand, they show a higher risk of severe side effects and notably increased risk of infection in the first months after a therapy pulse. On the other hand, a proportion of patients profit from disease stabilization and therapeutic effects persisting years beyond the end of therapy, inducing long-lasting therapy-free disease stability [10–12]. Substance-specific risk reduction strategies need to be applied. In comparison, conventional immunotherapies require continuous therapy with cumulative risks over time, counting towards the individual risk-benefit balance.

Considering the disease course in the long term, there is an advantage of using high-efficacy versus lower-efficacy DMTs from the beginning. This treatment strategy is supported by registry data, although prospective studies are lacking. Due to a likely increased risk for severe side effects and in consideration of individual life circumstances, the use of high-efficacy DMTs at the beginning of the disease should be decided individually, following the neurologist’s recommendations and the patient’s wishes.

Different therapy concepts exist within the group of high-efficacy DMTs. I) Sustained therapy: efficacy relatively immediate with application and accompanied by reversibility after discontinuation: natalizumab and S1P receptor modulators (as well as ocrelizumab and ofatumumab with limitation due to the mechanism of action), versus II) pulsed therapy: efficacy due to immune depletion and repopulation significantly beyond the half-life of the drug, possibly also permanent therapy-free disease stability: alemtuzumab and cladribine (and possibly ocrelizumab, with severe limitation due to mechanism of action).

Although the DGN guideline mentions chronic/continuous versus pulsed therapy approaches, it clearly prefers the chronic therapy approach. This is justified by pointing towards the lack of long-term data for pulsed therapies, which, however, is not correct. Ultimately, education about both therapy concepts must be provided, especially since the patient must be informed about the possibility of disease stabilization without continuous therapy [10].

## Question 3: when should the immunotherapy be terminated? Should the therapy generally be terminated after a few [5] years, or is long-term and sometimes permanent therapy feasible? To be considered in this context: the problem of disease reactivation/rebound

The scientific data on this clinically highly relevant question is scarce. The only available data result from retrospective observational studies mostly on older injectable MS therapeutics and comprising relatively small cohorts. A prospective paper from the Global MS Database describes that while relapse rates remain stable after discontinuation of injectable MS therapies, disease progression is significantly accelerated [13]. These results are consistent with smaller retrospective observations. So if treatment is well tolerated and safe, patients should be motivated to continue.

Special consideration must be given to agents inhibiting leukocyte migration (natalizumab and the S1P receptor modulators fingolimod, ozanimod, ponesimod, siponimod). Here, discontinuation without a concept for follow-up treatment should be the exception due to possible recurrence or even rebound of disease activity (challenging situations arise, for example, in the context of pregnancy, lactation, or surgery).

IRTs (alemtuzumab, cladribine, and within limitation maybe also ocrelizumab) are special cases, in the sense that disease stability without further or follow-up treatment is part of the therapeutic concept. Available data indicate that about 50% of patients can be stable for many years after alemtuzumab without a need for follow-up treatment, including the possibility of treating recurrent disease activity again with CD52 depletion (with the prospect of achieving re-stabilization).

Only a few controlled studies currently exist for the discontinuation of B-cell depleting drugs. However, a reversible mechanism of action, and therefore ultimately a return of disease activity, can be expected due to the B-cell dominance of the depletion principle. Established prognostic or diagnostic markers (such as the dynamics of depleting vs. repopulating immune cell types) that would indicate durable remission for a specified group do not exist for MS. Hence, also IRTs require established guidelines for monitoring and appropriate action plans for recurring disease activity.

More attention is now being paid to disease activity in relation to age, effects of therapy relating to age, and phenomena of immune senescence versus immunocompetence in old age. Roughly, the inflammatory activity and the effect of immunotherapy, especially the influence on progression, decrease with age. When weighing the therapeutic goals and benefit-risk profile, considering disease activity becomes more important, especially at a higher age (> 50).

We generally recommend that MS patients who are stable on a given DMT, receive clinical and/or radiological monitoring, and are without any safety or tolerability issues, should continue therapy. Discontinuing or pausing treatment is associated with the risk of recurrence of disease activity and/or progression, depending on the mechanism of action. Discontinuing or pausing treatment at a patient’s explicit request (without planned follow-up therapy) may be done if adhering to clear guidelines for clinical and imaging monitoring.

The DGN guideline positions itself very clearly towards the discontinuation of disease-modifying therapy in the long term and strongly recommends this possibility after 5 years. From the point of view of the MSTCG, this recommendation is not feasible considering the available data and can even create risks for some patients. Discontinuation or pausing of therapy at the explicit request of the patient (without a planned follow-up therapy) can take place under clear conditions for clinical and imaging monitoring but is by no means the rule. Corresponding discontinuation studies have only just been initiated internationally. On the other hand, there are clear epidemiological data on a possible “re-activation” in the sense of disease progression at any time, as well as data on a re-activation or rebound after discontinuation of immunomodulatory drugs. In addition, increased disease activity (rebound) may occur after discontinuation of natalizumab or S1P receptor modulators. In this line of critique, from the point of view of the MSTCG, the DGN guideline balances medication safety for the patient higher than modern options for long-term disease stabilization.

Several studies and reports describe the further development of MS and clinical and paraclinical disease activity after discontinuing DMT. The course after discontinuation depends on various factors such as disease severity in the individual patient, disease duration, comorbidities, and the type of DMT. While pulsed immunotherapies tend to stabilize disease over the longer term, maintenance therapies suggest a more rapid return of disease activity after cessation. The therapy sequence is also essential [14, 15]. In addition, there are immunopathogenic factors (genetics, environment, lifestyle).

Another factor to consider is differences in wash-out periods, i.e., the times between discontinuation of a substance and initiation of follow-up treatment (typically from one to six months). Special consideration in this context is given to drugs that affect leukocyte migration [16, 17]. For them, in addition to the expected recurrence of disease activity due to discontinuation, various reports describe a rebound, meaning a return of disease activity to a level exceeding that before the start of therapy. Although numerous studies describe this effect for fingolimod and natalizumab, rebound does not occur in every individual after discontinuing these therapies. However, an appropriate follow-up treatment should always be administered after fingolimod and natalizumab to prevent the potential recurrence of disease activity.

Hence, discontinuation or suspension of a medication for the therapy of (highly) active MS, either based on suboptimal efficacy or safety concerns, must be accompanied by a clear follow-up concept. The following factors should be considered when selecting a follow-up medication: 1.) disease activity (clinical and MRI): the higher the disease activity, the larger the need for immediate initiation of a new therapy; 2.) disease severity; 3.) half-life as well as biological activity of the previous medication (differentiation between so-called maintenance therapies (natalizumab, S1P receptor modulators, partly ocrelizumab) and pulsed therapies, (alemtuzumab, cladribine, partly ocrelizumab)); 4.) the risk of “carry-over” PML should be reduced as much as possible, and clinical, MRI, and liquid diagnostic parameters (detection of HPyV-2 [JCV] DNA by PCR) should be used to determine the baseline or pre-conversion status. The risk of recurrence of disease activity or rebound (especially after leukocyte migration therapies such as natalizumab or S1P receptor modulators) should be considered and can be expected 2–6 months after discontinuation of these agents.

For SPMS, uncertainties may arise further down the line when patients with initially active disease have been treated and no longer have relapses. Various experts, including the authors of the North American guideline, recommend discontinuing therapy when there is pure progression without relapse. However, it is unclear whether the DMT suppresses relapse activity despite not affecting progression, meaning that patients would continue to benefit from the relapse rate reduction provided by DMT. If therapy is discontinued in SPMS patients, close monitoring of subsequential inflammatory activity is essential.

## Question 4: how strongly should aspects specified in the official therapy approval be reflected in a treatment recommendation? To be considered in this context: is the equivalent recommendation for rituximab (off-label use) and ocrelizumab as approved B-cell-depleting therapy (on-label) appropriate?

Drugs are approved by regulatory agencies following a detailed and rather rigid process. Approvals of therapies are associated with distinct “labels”. Regarding MS therapies, labels are usually related to the populations that have been tested and for whom a positive benefit-risk ratio can be assumed. Formally this means the approved drugs can be used under the prerequisites and conditions provided within the label – a medicolegal aspect that must not be neglected.

Guidelines certainly are asked to put those approval texts into a given context and a disease treatment concept. However, guidelines should not a priori conflict with the liberty of treatment choice nor create medicolegal conflict situations by recommending off-label therapies where on-label therapies are available.

With regard to the DGN guideline, the most relevant problems are generated by the expressed preference for the “escalation approach” and the categorization of drugs into three classes of efficacy. This stands in conflict with the scientific basis of the effectiveness/efficacy classes (see question 2) and the approval texts.

In recent years, anti-CD20 antibodies have become established as a further therapeutic option for relapsing MS. Ocrelizumab has been approved since 2018 and was developed from rituximab, which was never formally approved for MS treatment (only off-label use). Ofatumumab, which is administered subcutaneously, received approval in March 2021.

A critical problem is generated by the potential off-label use of the CD20 antibody rituximab, based on the assumed similarity of the molecular target. Rituximab is not approved for MS, despite broad off-label use in various countries worldwide, but is placed on an “equal footing” with other preparations for B-cell therapy (ocrelizumab, ofatumumab). This is problematic since there are no available class I evidence study results to formally allow this statement for rituximab. While on the other hand, ocrelizumab and ofatumumab have provided strong class I evidence from phase III trials studying the efficacy and safety of these CD20 antibodies in a head-to-head comparison (OPERA, ORATORIO, ASCLEPIOS trials).

Ocrelizumab was also the first compound ever to be approved for PPMS. In the relatively small but well-structured pivotal study, a significant delay in disability progression since the onset of progressive MS, particularly in the first year after initiation of therapy, was achieved in patients under 50 years of age with active disease and short disease duration. This resulted in a theoretical delay of wheelchair use by up to seven years [18]. The pivotal study indicates better efficacy in younger patients with shorter disease duration [19]. For PPMS, ocrelizumab is thus the only currently approved treatment. Even if the effect in PPMS is comparatively small as measured by the EDSS, at least younger patients benefit from therapy with ocrelizumab, especially since there is no approved alternative. There are no data from controlled trials in older patients (> 55 years) with a longer disease course (> 15 years) and a higher degree of disability (EDSS score > 6.5). Nevertheless, according to the authors’ assessment, therapeutic nihilism should not be practiced here. In particular, for patients at risk of losing physical independence, a therapeutic attempt is justified. For the patient’s quality of life, this attempt can be decisive.

Ocrelizumab and especially ofatumumab are “next-generation” antibodies with less or no murine compounds, therefore further reducing immunogenicity. Studies to inform about vaccination immune responses in MS patients have been carried out in ocrelizumab [20].

While the authors agree that similar efficacy is possible with rituximab if studied under controlled conditions, it is formally off-label and thus leads to medicolegal problems. In this context, the MSTCG points out the importance of providing treatment recommendations in principle in line with the approval texts. Off-label therapy should clearly be possible in case of insufficient alternatives or situations where on-label therapies are not sufficient to control the situation.

The same argumentation holds for the four available S1P receptor modulators, for which one could say they are all the same active group but that all ultimately have distinct approvals. This is partly due to the specific studies (fingolimod in RRMS, siponimod in SPMS) but also due to the approval history (the first S1P receptor modulator fingolimod was only approved as a second-line therapy to ensure safety as best as possible, which was ultimately not a wrong initial decision). However, one cannot say that all S1P receptor modulators are the same, but we must look at the specific approval situations.

The MSTCG has approached this problem by considering the formal approval texts while at the same time dividing the therapy scheme with the more modern and appropriate subdivisions of relapsing-remitting MS versus progressive MS (Fig. 1). This tactic guarantees maximum freedom of therapy on the one hand and, on the other hand, also allows classification and, if necessary, therapy sequence.

A guideline cannot be a “recipe” for all aspects of a complex and, on an individual level adaptive, decision process.

## Supplementary Information

**Additional file 1.** Conflicting interests for all authors.

## Data Availability

Not applicable.
