# Supplementary material for: Multiple sclerosis therapy consensus group (MSTCG): answers to the discussion questions
Source: Neurol Res Pract. 2021 Aug 6;3:44. doi: 10.1186/s42466-021-00140-1 (PMC8344158; doi:10.1186/s42466-021-00140-1)
Supplement: Supplementary file 1 — Additional file 1. Conflicting interests for all authors. [file 42466_2021_140_MOESM1_ESM.docx]

**Competing interests inquiry for article NRAP-D-21-00091R0**

HW: Received grants or contracts from BMBF, Novartis, Genzyme, Roche, Merck, Biogen, DFG, European Union; Royalties or licenses from Hogrefe, Springer Medizin; Consulting fees from Merck, Novartis, Roche, Sanofi; Payment for expert testimony AXA banque patrimoniale, Dialectica, Swiss MS society, Peervoice; Support for attending meetings and/or travel from Biogen, FEO, Merck, Novartis, Pfizer, Sanofi, Celgene, Excell/Bial, Roche. Participation on a Data Safety Monitoring Board or Advisory Board for PSI CRO Germany.

RG: Received grants or contracts from Novartis, Biogen, Sanofi; Consulting fees from Merck-Serono, Janssen, Biogen, Novartis, Sandoz; Payment or honoraria for lectures, presentations, speakers bureaus, manuscript writing or educational events from BayerVital, Biogen, Eisai, Bristol Myers Squibb, TEVA, Merck-Serono, Novartis, Roche, Sanofi-Aventis. Participation on a Data Safety Monitoring Board or Advisory Board for Roche, Genentech; Leadership or fiduciary role in other board, society, committee or advocacy group, paid or unpaid as Chair of DMSG Medical Advisory Board (honorary position). Has stock or stock options for Roche, Bayer, Merck.

RG is the Editor-in-Chief of Therapeutic Advances in Neurological Disorders, therefore, the peer review process was managed by alternative members of the board and the submitting editor was not involved in the decision-making process.

FZ: Participation in a scientific advisory board or lecturing activity for Novartis, Roche, Celgene, Janssen, Novartis Pharma, Janssen Pharmaceutica, Sandoz International, F. Hoffmann–La Roche; Research projects/conduction of clinical studies for Precisis, UCB, SK Life Science, Biogen, Grünenthal, Actelion. Ownership interests in the healthcare sector i.e., Patent application Cytokine derivatives.
